# Genome-wide association study of antisocial personality disorder diagnostic criteria provides evidence for shared risk factors across disorders

**DOI:** 10.1097/YPG.0000000000000352

**Published:** 2023-09-19

**Authors:** Wenqianglong Li, Hang Zhou, Johan H. Thygesen, Mathis Heydtmann, Iain Smith, Franziska Degenhardt, Markus Nöthen, Marsha Y. Morgan, Henry R. Kranzler, Joel Gelernter, Nicholas Bass, Andrew McQuillin

**Affiliations:** a Molecular Psychiatry Laboratory, Division of Psychiatry, University College London, London, UK; b Department of Psychiatry, Yale School of Medicine, New Haven; c Department of Psychiatry, Veterans Affairs Connecticut Healthcare System, West Haven, Connecticut, USA; d Institute of Health Informatics, University College London, London, UK; e Royal Alexandria Hospital, NHS Greater Glasgow and Clyde, Paisley, UK; f Department of Gastroenterology, Dumfries & Galloway Royal Infirmary, Cargenbridge, Dumfries, Scotland; g Substance misuse service, Mayfield Centre, St Ninians, Stirling, UK; h Department of Child and Adolescent Psychiatry, University of Duisburg-Essen, Essen; i Institute of Human Genetics, University of Bonn, School of Medicine & University Hospital Bonn, Bonn, Germany; j UCL Institute for Liver & Digestive Health, Division of Medicine, Royal Free Campus, University College London, London, UK; k Department of Psychiatry, University of Pennsylvania Perelman School of Medicine; l Crescenz Veterans Affairs Medical Center, Philadelphia, Pennsylvania; m Departments of Genetics and Neuroscience, Yale University School of Medicine, New Haven, Connecticut, USA

**Keywords:** alcohol misuse, comorbidity, genetic, personality disorders

## Abstract

**Introduction:**

While progress has been made in determining the genetic basis of antisocial behaviour, little progress has been made for antisocial personality disorder (ASPD), a condition that often co-occurs with other psychiatric conditions including substance use disorders, attention deficit hyperactivity disorder (ADHD), and anxiety disorders. This study aims to improve the understanding of the genetic risk for ASPD and its relationship with other disorders and traits.

**Methods:**

We conducted a genome-wide association study (GWAS) of the number of ASPD diagnostic criteria data from 3217 alcohol-dependent participants recruited in the UK (UCL, *N* = 644) and the USA (Yale-Penn, *N* = 2573).

**Results:**

We identified rs9806493, a chromosome 15 variant, that showed a genome-wide significant association (*Z*-score = −5.501, *P* = 3.77 × 10^−8^) with ASPD criteria. rs9806493 is an eQTL for SLCO3A1 (Solute Carrier Organic Anion Transporter Family Member 3A1), a ubiquitously expressed gene with strong expression in brain regions that include the anterior cingulate and frontal cortices. Polygenic risk score analysis identified positive correlations between ASPD and smoking, ADHD, depression traits, and posttraumatic stress disorder. Negative correlations were observed between ASPD PRS and alcohol intake frequency, reproductive traits, and level of educational attainment.

**Conclusion:**

This study provides evidence for an association between ASPD risk and SLCO3A1 and provides insight into the genetic architecture and pleiotropic associations of ASPD.

## Introduction

Antisocial personality disorder (ASPD) is characterized by traits of antagonism and disinhibition. Specific traits within these domains include manipulativeness deceitfulness, recklessness, and lack of empathy. These traits often arise in childhood or early adolescence and continue into adulthood (APA, 2000). ASPD is associated with adverse outcomes not only for the person living with the disorder, but also for their families and friends and for wider society (NICE, Excellence C, 2014). The prevalence estimates of ASPD vary considerably, from 1 to 6.8% in men and from 0.2 to 1% in women (Torgersen *et al.*, 2001; Coid *et al.*, 2006), with the higher prevalence in men being a consistent finding (Glenn *et al.*, 2013). The Diagnostic and Statistical Manual of Mental Disorders (fourth edition, DSM-IV) diagnostic criteria for ASPD include symptoms and signs of conduct disorder in childhood and difficulties with cognition, affectivity, interpersonal functioning and impulse control in adulthood (APA, 2000).

Epidemiological studies indicate that both genetic and environmental factors influence the development of ASPD. Ferguson *et al*. (Ferguson, 2010), undertook a meta-analytical review of antisocial personality and behaviour that covered a range of personality traits and antisocial behaviour itself. The results showed that 56% of the variance in antisocial personality and behaviour could be explained by genetic influences, while shared and unique environmental factors, including non-genetic biological factors such as trauma and non-family socialization, explained the remainder.

Genome-wide association studies (GWASs) have been used to identify common genetic risk variants in psychiatric disorders (Ripke *et al*., 2014). To date, three GWASs have been undertaken to identify association with antisocial behaviour including ASPD. Tielbeek *et al*. reported the first GWAS of adult antisocial behaviour using a community sample of twin pairs registered with the Australian Twin Registry and their families comprising 4816 individuals from 2227 independent families; the definition of adult antisocial behaviour was based largely on DSM-IV criteria (Tielbeek *et al*., 2012). No genome-wide significance for association with antisocial behaviour was identified. Rautiainen *et al*. (Abram *et al.*, 2015) conducted a discovery GWAS in 370 Finish criminal offenders who fulfilled DSM-IV criteria for a diagnosis of ASPD and 5850 general population controls (Rautiainen *et al*., 2016). None of the associations reached genome-wide significance in this analysis; however, eight suggestive variants associated with ASPD originated in the vicinity of *HLA-DRA* on chromosome 6. These SNPs were genotyped in a replication cohort of 173 offenders and 3766 controls and the results from both cohorts were meta-analyzed. One SNP, rs4714329, on chromosome 6p21.2 close to *LINC00951* (*Long Intergenic Non-Protein Coding RNA 951*) was associated with ASPD at genome-wide significance (*P* = 1.6 × 10^−9^). More recently Tielbeek *et al*. reported a genome-wide significant association with SNPs at the *FOXP2* (Forkhead Box Protein P2) locus and a broadly defined antisocial behaviour phenotype in data from 28 discovery samples involving 85 359 participants and five independent replication samples involving 8058 participants (Tielbeek *et al.*, 2022).

ASPD displays considerable comorbidity with other psychiatric diseases (Abram *et al.*, 2015; Tielbeek *et al.*, 2018b). Thus individuals with ASPD have been shown to be at high risk for substance use disorders such as alcohol dependence (Yoshino *et al.*, 2000; Bahlmann *et al.*, 2002); depression (Moody *et al.*, 2016), attention deficit hyperactivity disorder (ADHD) (Anney *et al.*, 2009; Instanes *et al.*, 2016), anxiety disorder (Galbraith *et al.*, 2014; Brook *et al.*, 2016), post-traumatic stress disorder (PTSD) (Goodwin and Hamilton, 2003), and schizophrenia (Schiffer *et al*., 2017; Sedgwick *et al.*, 2017). The rate of alcohol use disorder in people with ASPD is particularly high at an estimated 76.7% (Guy *et al.*, 2018). Moreover, data from two consecutively collected cohorts of prisoners has shown that alcohol dependence syndrome (ADS) shows high comorbidity with ASPD, suggesting that there may be common biological risk mechanisms (Yoshino *et al.*, 2000; Bahlmann *et al.*, 2002). Malone *et al*. (2004) found a significant but moderate genetic influence on adult antisocial behaviour and ADS at ages 17, 20, and 24 years in a cross-sectional twin study. Moreover, Malone *et al*. (Malone *et al.*, 2004) showed, using cross-twin cross-trait correlations, that the covariation of antisocial behaviour and ADS was due to genetic factors, and that both disorders have a common genetic vulnerability, suggesting they might share susceptibility genes. However, Tielbeek *et al*. (2018a) assessed the relationships between antisocial behaviour risk and substance use disorder risk and identified significant genetic correlations with cannabis use and smoking but not with alcohol consumption. However, the nature of any common genetic susceptibility has yet to be discovered.

The aim of the present study was to use genome-wide data to examine the genetic architecture of ASPD symptoms and to identify potential genetic risk factors. By using a cohort of people clinically diagnosed with alcohol dependence syndrome for whom there was also data for ASPD diagnostic criteria, we reduced potential confounding of the genetic risk for alcohol dependence syndrome and environmental exposure to alcohol. We also estimated the genetic correlation of ASPD diagnostic criteria with other complex traits.

## Methods

### Participants

The UCL cohort

A total of 644 participants were recruited from a variety of UK community and hospital-based services providing support and treatment for individuals with alcohol dependence. All participants had received a clinical diagnosis of alcohol dependence according to ICD-10 (F10.2). A clinical diagnosis of alcohol dependence was confirmed by clinicians and trained researchers using the Alcohol Dependence Syndrome section of the Semi-Structured Assessment for the Genetics of Alcoholism II questionnaire (Bucholz *et al*., 1994). The assessment allowed the diagnosis of ASPD to be made as a DSM-IV binary trait but also allowed for the generation of a quantitative ASPD criterion score (American Psychiatric Association, 2000). All participants were of English, Scottish, Welsh, or Irish descent with a maximum of one grandparent of non-British (but still Western European) ancestry; none of the individuals was related. Approval for the study was obtained from the NHS Metropolitan Multi-centre Research Ethics Committee (now the South Central - Hampshire A Research Ethics Committee) approval number MREC/03/11/090. All participants provided signed informed consent.

#### The Yale-Penn cohort

Participants were recruited as part of the Yale-Penn study of the genetic bases of drug and alcohol dependence, as described elsewhere (Kember *et al*., 2023). The subjects were interviewed using the Semi-Structured Assessment for Drug Dependence and Alcoholism (Pierucci-Lagha *et al*., 2005). Lifetime psychiatric and substance use disorders were diagnosed based on DSM-IV criteria. A European American (EA) subset of subjects from the Yale-Penn dataset were included in the current study. These included 1081 from Yale-Penn Phase 1, 1029 in Yale-Penn Phase 2, and 463 in Yale-Penn Phase 3. The study was approved by the Institutional Review Boards at the sampling sites and written informed consent was obtained from all study participants. Certificates of confidentiality were issued by the National Institute on Drug Abuse and the National Institute on Alcohol Abuse and Alcoholism.

### Phenotypes

#### The DSM-IV criteria for ASPD include

A: Evidence of conduct disorder (15 criteria) with onset before age 15; B: evidence of ASPD (9 criteria) that is a pervasive pattern of disregard for, and violation of, the rights of others occurring since age 15 years (see Supplementary Material 1, Supplemental digital content 1, http://links.lww.com/PG/A312 for the ASPD diagnostic criteria). A count of fulfilled ASPD diagnostic criteria rather than ASPD diagnosis was used as the phenotype to maximize the informativeness of the data. For each participant positive criteria were summed. Participants were excluded if their behaviour occurred during schizophrenia/manic episodes.

### Genotyping and quality control

Genome-wide genotyping of genomic DNA from the UCL cohort was undertaken using the Illumina PsychArray. Genomic DNA from the Yale-Penn cohort underwent genotyping in three phases using the Illumina HumanOmni1-Quad array (phase 1), the Illumina HumanCore Exome array (phase 2), the Illumina Multi-Ethnic Genotyping array (phase 3), and each phase was analysed separately. Details of the genotyping, pre-imputation quality control and imputation are provided in the Supplement.

### Statistical analysis

#### Association tests

GWAS analyses of the data generated in the four cohorts were conducted separately on imputed best-guess genotypes using a linear regression model with a quantitative scale of ASPD diagnostic criteria as the phenotype, and sex, age, and the first 10 principal components as covariates. The analyses in the UCL data were performed in PLINK2 (Chang *et al.*, 2015) while the separate analyses of the three Yale-Penn data sets (which include relatives) were performed in GEMMA-v0.98.1 (Zhou and Stephens, 2012). Analysis of chromosome X data was performed using XWAS (version 3.0) with male genotypes on the X chromosome were coded as 0/2 (Gao *et al*., 2015).

#### Meta-analysis of four cohorts

Sample size weighted meta-analysis of the four ASPD GWAS data sets including chromosome X (N = 3217) was performed using METAL (Willer *et al.*, 2010).

#### Fine mapping

LocusZoom was used to make local association plots by uploading the meta-analysis summary statistics (Teslovich *et al*., 2010). Hg19/1000 Genomes Nov 2014 EUR was used for the background LD structure.

#### Gene-based test, pathway, and enrichment analyses

Functional Mapping and Annotation of Genome-Wide Association Studies (FUMA) software was used to explore gene prioritization, gene expression, pathway process enrichment with meta-analysis summary statistics as input. FUMA implemented Bonferroni correction (*P*_bon_ < 0.05) to correct for multiple testing (Watanabe *et al.*, 2017).

#### Phenome-wide association analysis

To examine whether any of the top hits and related genes identified in the present study are associated with other complex traits, phenome-wide association analysis (PheWAS) plots were created by exploring the 4756 GWAS summary stats available on the GWAS ATLAS platform (Watanabe *et al*., 2019). All GWAS SNPs and related genes were used in the analysis. SNPs with *P*-value <0.05 were used in the PheWAS SNP plot. *P*-values were adjusted for multiple comparisons using Bonferroni correction.

#### Polygenic risk scores

Polygenic risk score (PRS) analyses were performed to test whether risk alleles for a variety of psychiatric and behavioural traits correlated with genetic risk variants associated with the ASPD diagnostic criteria scores in the UCL and Yale-Penn samples. PRSice2 was used to estimate the best-fit PRS at a range of *P*-value thresholds (Choi *et al.*, 2020). The linkage disequilibrium (LD) threshold was set to an R^2^ of 0.1 and a distance of 250 kb.

The meta-analysis of the PRS results from UCL and Yale-Penn was conducted using metagen in the meta package in R (Balduzzi *et al.*, 2019). The FDR method was used to correct for multiple comparisons.

Summary statistics of complex traits and psychiatric disorders were downloaded from publicly available resources [Psychiatric Genomics Consortium (PGC): https://www.med.unc.edu/pgc/results-and-downloads and the GWAS ATLAS: http://atlas.ctglab.nl]. The summary statistics for a GWAS of coronary artery disease was also included to act as a negative control for the PRS analyses (Nikpay *et al*., 2015).

## Results

A total of 3217 individuals with a lifetime history of alcohol dependence syndrome and an ASPD diagnostic criterion score were included in the study (Table 1). There were no significant differences in the sex distribution of age between the UCL and Yale-Penn cohorts but the UCL cohort were less severely affected (*P* < 0.01) (Table 1).

**Table 1:** Demographics and total ASPD diagnostic criteria counts for the GWAS cohorts

Sample (n)	Sex (% men)	Age, mean (range)		ASPD diagnostic criteria score (mean)	
		Men	Women	Men	Women
UCL (644)	67	44 (19–74)	45 (22–69)	4	2
Yale-Penn Phase 1 (1081)	63	40 (16–71)	39 (16–69)	7	6
Yale-Penn Phase 2 (1029)	67	40 (18–76)	39 (19–80)	7	6
Yale-Penn Phase 3 (463)	68	40 (17–73)	38 (18–75)	7	5
Total	63	41	40	7	5

### GWAS of the four cohorts and meta-analysis

#### Results from the individual cohorts

We found a genome-wide significant locus on chromosome 14q.13.1 (rs142893681, *P* = 3.19 × 10^−8^), in the vicinity of the *SNX6* (sorting nexin 6) gene in the UCL cohort GWAS. A genome-wide significant locus on chromosome 18q11.2 (rs59381075, 1.98 × 10^−8^), near the *ZNF521* (Zinc Finger Protein 521) gene was found in the Yale-Penn Phase 1 GWAS. No genome-wide significant associations were identified in the Yale-Penn Phases 2, 3 cohorts or on chromosome X (Supplementary Tables 1–4. Supplemental digital content 2, http://links.lww.com/PG/A313).

#### Results from meta-analyses

The meta-analysis of the four individual GWAS of ASPD criteria scores identified a genome-wide significant association on chromosome 15q.26.1 (rs9806493 *P* = 3.77 × 10^−8^) near to *SLCO3A1* (Solute Carrier Organic Anion Transporter Family Member 3A1) (Table 2 and Supplementary Figure 1, Supplemental digital content 1, http://links.lww.com/PG/A312). Two additional independent SNPs showed suggestive evidence of association, rs10186418 (*P* = 2.79 × 10^−7^) in an intron of in *KCNS3* (Potassium Voltage-Gated Channel Modifier Subfamily S Member3) and rs11682196 (*P* = 3.69 × 10^−7^) in an intron of *CTNNA2* (Catenin Alpha 2). The two SNPs, that were identified at genome-wide significance (rs142893681 and rs59381075) (*P* < 5 × 10^−8^) in the UCL and Yale-Penn Phase 1 individual GWASs respectively, were NS in the meta-analysis. No significant results were found from the meta-analysis of the X chromosome data.

**Table 2:** Top independent variants associated with ASPD in the meta-analysis of the GWAS data from the UCL and Yale-Penn cohorts

CHR	SNP ID^a^	Effect allele	Other allele	Gene	Weight	Z-score	*P*-value	Effect allele frequency (%)	Direction
15	rs9806493	C	T	*SLCO3A1*	1673	−5.501	3.77 × 10^−8^	47.1	––??
2	rs10186418	A	G	*KCNS3*	3197	5.137	2.79 × 10^−7^	86.8	++++
2	rs11682196	C	A	*CTNNA2*	3157	−5.084	3.69 × 10^−7^	86.9	––––
7	rs967758	C	T	*Y_RNA*	2153	−4.868	1.13 × 10^−6^	20.2	––?–
20	rs6076184	T	C	*RP5-1100I6.1*	2181	4.807	1.53 × 10^−6^	5.3	++?+
1	rs6691165	C	A	*MIR552*	3175	−4.789	1.68 × 10^−6^	45.2	––––

Gene is the gene located closest to the lead SNP; or where there are multiple genes in the region the gene for which the SNP has the most deleterious annotation in ANNOVAR. Direction: - for negative, + for positive and? for missing in the UCL, Yale-Penn Phases 1, 2, and 3 samples respectively. The SNP marked in bold text reached a genome-wide level of significance in the meta-analysis.

CHR, chromosome; SNP, single nucleotide polymorphism.

a Only SNPs that were present in both UCL and one or more Yale-Penn samples are shown.

### Fine mapping and expression quantitative trait loci

**Fig. 1:**
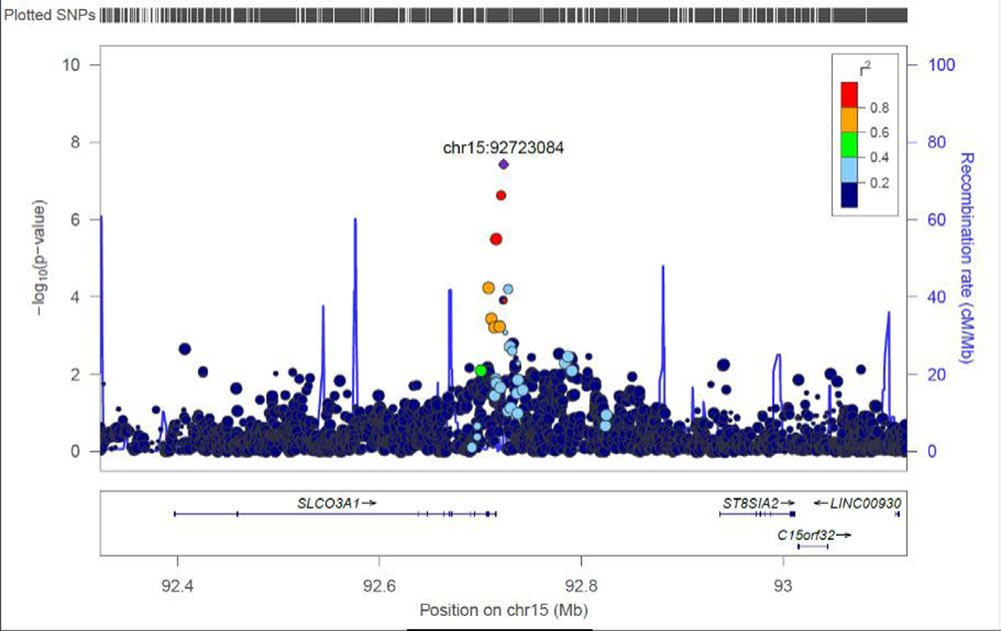
Regional locus plot of the association findings with rs9806493 close to the *SLCO3A1* gene in the meta-analysis of ASPD diagnostic criteria scores.

rs9806493 is located 7.5 kb downstream from the *Solute Carrier Organic Anion Transporter Family Member 3A1* (*SLCO3A1*) gene (Fig. 1). All of the available SNPs in linkage disequilibrium with rs9806493 (R^2^ > 0.2) map to a region towards the 3’ end of *SLCO3A1*. rs9806493 is identified as a peripheral blood cis-expression quantitative trait loci (cis-eQTL) for *SLCO3A1* (Z-score = −9.33, *P* = 1.09 × 10^−20^) in the eQTLGen database (Võsa *et al*., 2021). It is also identified as an eQTL for *SLCO3A1* (regression slope = −0.035, *P* = 1.01 × 10^−4^) in the QTL maps from the PsychEncode project (Gandal *et al*., 2018). However, it is not identified as an eQTL for *SLCO3A1* in the Genotype-Tissue Expression (GTEx) database (version 8.0) (Consortium, 2020).

### Gene-based tests, pathway, and enrichment analyses

MAGMA gene-based, gene-set tests, and tissue expression analysis of the individual UCL and the Yale-Penn GWAS did not identify any statistically significant findings. MAGMA gene-based and tissue expression analyses of the data from the GWAS meta-analysis showed no evidence for association with ASPD. MAGMA gene-set tests of the meta-analysis data provided nominal evidence for association of several gene sets with ASPD. However, none of these survived Bonferroni correction for multiple testing (Supplementary Tables 5–10. Supplemental digital content 2, http://links.lww.com/PG/A313)

### Phenome-wide association analysis

**Fig. 2:**
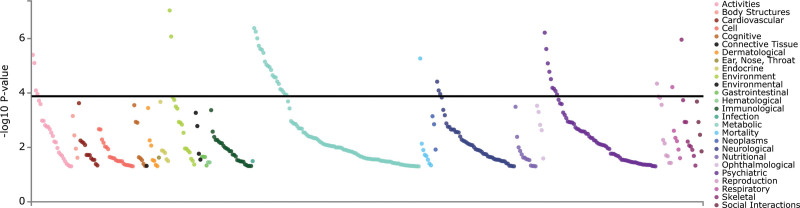
*SLCO3A1* gene PheWAS plot. Phenome-wide association analysis for the *SLCO3A1* gene from 436 GWASs. The results were sorted by domain and *P*-value. The Bonferroni corrected *P*-value threshold is 1.15 × 10^−4^ (horizontal black line). The studies and phenotypes examined that report an association that survived Bonferroni correction are shown in Supplementary Table 10. Supplemental digital content 2, http://links.lww.com/PG/A313.

PheWAS analyses using the GWAS Atlas platform were performed to examine secondary phenotypes associated with rs9806493 and the *SLCO3A1* gene. rs9806493 was associated with the reproductive (and risk-taking) trait: “number of sexual partners” (*P* = 0.0003, Bonferroni corrected *P* = 0.00049; Supplementary Figure 2, Supplemental digital content 1, http://links.lww.com/PG/A312 Supplementary Table 11. Supplemental digital content 2, http://links.lww.com/PG/A313). The *SLCO3A1* gene was associated with 43 different traits after Bonferroni correction, including educational attainment (*P* = 9.28 × 10^−8^), BMI (*P* = 5.56 × 10^−7^), broad depression (*P* = 6.06 × 10^−7^), seeing a doctor for nerves, anxiety, tension or depression (*P* = 2.42 × 10^−6^), alcohol dependence (*P* = 1.66 × 10^−5^), and depression (*P* = 1.13 × 10^−4^; Table 3 and Fig. 2, and Supplementary Table 12, Supplemental digital content 2, http://links.lww.com/PG/A313).

**Table 3:** Top significant trait associations in the *SLCO3A1* phenome-wide association analysis

Trait	Domain	Reference	*P*-values	Participants (n)
Educational attainment	Environment	Okbay*et al.* (2016)	9.28 × 10^−8^	328 917
Broad depression	Psychiatric	Howard *et al.* (2018)	6.06 × 10^−7^	322 580
Educational attainment	Environment	Lee *et al.* (2018)	8.40 × 10^−7^	766 345
Seen doctor (GP) for nerves, anxiety, tension or depression	Psychiatric	Watanabe *et al.* (2019)	2.42 × 10^−6^	383 771
Alcohol dependence	Psychiatric	Wang *et al.* (2013)	1.66 × 10^−5^	2322
Lifetime number of sexual partners	Reproduction	Watanabe *et al.* (2019)	4.52 × 10^−5^	316 569
Depression	Psychiatric	Howard *et al.* (2019)	1.13 × 10^−4^	500 199

A total of 436 GWAS were included in the PheWas., the Bonferroni corrected *P*-value threshold is 1.15 × 10^−4^.

### Polygenic risk score analysis

**Fig. 3:**
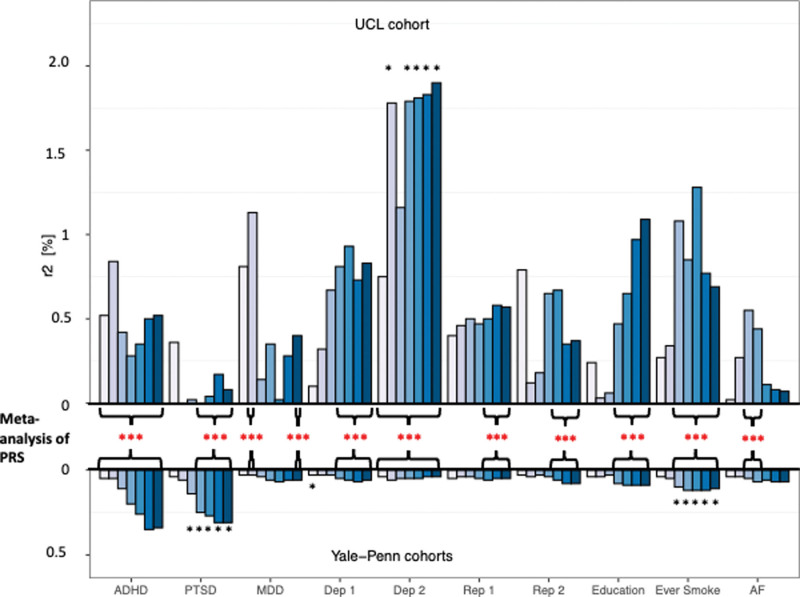
PRS analysis in the UCL and Yale-Penn ASPD samples. PRS results are shown at different *P*-value thresholds for each trait along with the percentage of variance explained by the PRS (Nagelkerke’s R^2^) for ten traits in the UCL and Yale-Penn cohorts. *Comparisons significant after FDR correction in the individual PRS analyses (black *) or in the PRS meta-analysis (red*). ADHD, attention deficit hyperactivity disorder; AF, alcohol frequency; Dep 1, depressive symptoms; Dep 2, seen doctor for nerves, anxiety, tension or depression; Education, age completed full-time education; Ever Smoke, Whether a participant had ever smoked a cigarette; MDD, major depression disorder; PTSD, post-traumatic stress disorder; Rep 1, age at first live birth (female); Rep 2, age at first birth (male).

**Fig. 4:**
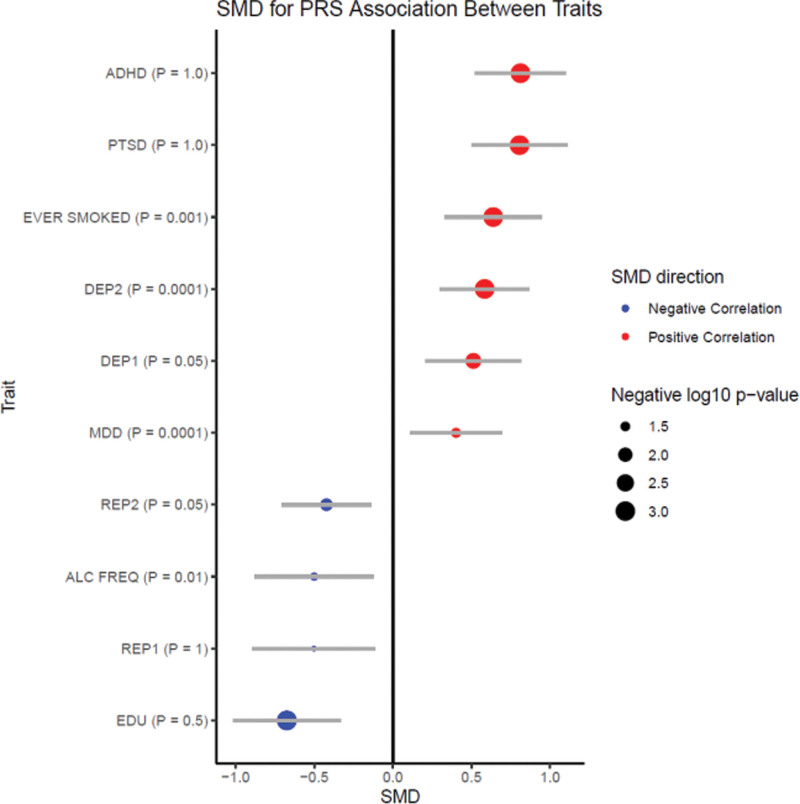
Polygenic risk score relationships among ASPD and other traits. The direction of correlations between ASPD PRS and the PRS for other traits are shown as the standardized mean difference (SMD). Redpoint estimates indicate a positive correlation; blue point estimates indicate a negative correlation. The size of the point estimates reflects the FDR adjusted -log_10_ of the *P*-value. ADHD, attention deficit hyperactivity disorder; PTSD, post-traumatic stress disorder, depression traits include: major depression disorder (MDD), depression symptoms and seen doctor for nerves, anxiety, tension or depression, reproduction traits include: age at first birth (female), age at first birth (male). See Supplementary Figure 3, Supplemental digital content 1, http://links.lww.com/PG/A312 for GWAS ATLAS genetic correlation results.

PRS analyses were performed to investigate the genetic correlation between major psychiatric disorders and other complex behavioural traits with ASPD diagnostic criterion scores (Figs. 3 and 4 and Supplementary Table 13. Supplemental digital content 2, http://links.lww.com/PG/A313). The analyses were performed using publicly available GWAS summary statistics downloaded from the PGC or the GWAS ATLAS (Supplementary Table 14. Supplemental digital content 2, http://links.lww.com/PG/A313).

The meta-analysis of PRSs results for educational attainment, alcohol intake frequency, and the reproductive traits ‘*age at first live birth in women and men’* were negatively correlated with higher ASPD criterion scores (*P*_FDR corrected_ < 0.05). The PRS for whether a subject had ever smoked, had depression including major depressive disorder, and two sub-clinical depressive traits were positively correlated with the number of ASPD diagnostic criteria (*P*_FDR corrected_ < 0.05).

The PRS for post-traumatic stress disorder did not show a consistent direction of effect in the UCL sample but was positively correlated with ASPD diagnostic criteria in the Yale-Penn cohorts (Table 4). None of the results from the PRS analyses with schizophrenia, anxiety, aggression, or coronary artery disease survived correction for multiple testing in the meta-analysis.

**Table 4:** PRS analysis in the UCL and Yale-Penn ASPD cohorts, and the meta-analysis results

Directions	Traits	Best *P*-value threshold		R^2^		Significance (P)		Significance (P meta-analysis)
		UCL	Yale-Penn	UCL	Yale-Penn	UCL	Yale-Penn	
Negative correlation	Age at first live birth (female)	0.5	1 × 10^−5^	0.58%	0.05%	0.038	> 0.05	0.044
	Age at first live birth (male)	1 × 10^−5^	1	0.79%	0.08%	0.015	0.01	0.018
	Age at last birth (female)	1 × 10^−5^	1	0.36%	0.08%	> 0.05	0.014	> 0.05
	Age of completion of full-time education	1	0.5	1.10%	0.09%	0.004	0.006	9.4 × 10^−4^
	Alcohol intake frequency	0.001	1	0.55%	0.07%	0.043	0.039	0.039
Positive correlation	Ever smoked	0.05	0.01	1.28%	0.12%	0.002	5.8 × 10^−4^	9.4 × 10^−4^
	MDD	1 × 10^−4^	0.05	1.13%	0.07%	0.004	0.036	0.03
	Depressive symptoms	0.05	0.5	0.93%	0.07%	0.009	0.036	0.006
	Seen doctor for nerves, anxiety, tension or depression	1	1 × 10^−4^	1.90%	0.06%	1.7 × 10^−4^	> 0.05	1.6 × 10^−3^
	PTSD	0.05	1 × 10-5	0.36%	0.31%	> 0.05	1.0 × 10^−9^	9.4 × 10^−4^

MDD, major depression disorder; PTSD, post-traumatic stress disorder.

*P*-values for the meta-analysis of PRS were corrected for multiple comparisons using FDR method.

## Discussion

The present study investigated the genetic architecture of ASPD criteria in the context of alcohol dependence and is to the best of our knowledge the largest meta-analytic GWAS of comparable clinical measures of ASPD undertaken to date. This GWAS meta-analysis identified a novel genome-wide significant signal with rs9806493 on chromosome 15q26.1 close to *SLCO3A1*. This marker is supported by additional SNPs in linkage disequilibrium with the main finding that did not reach genome-wide significance. In the PRS analysis, genetic correlations that survived correction for multiple testing were identified with genetic risk variants for many complex behavioural traits and psychiatric disorders including education attainment, smoking, alcohol intake frequency, reproductive behaviours, depression, PTSD, and ADHD.

Two of three eQTL databases indicate that rs9806493 is an eQTL for *SLCO3A1*. The ASPD risk allele in our study (rs9806493:T, note that the Z-score with allele C is negative; Table 2), is associated with increased expression of *SLCO3A1*.

*SLCO3A1* has also been shown to mediate the transport of Na (+)-independent of organic anions and hormones including thyroxine and vasopressin, the cyclic oligopeptides BQ-123 (endothelin receptor antagonist), and prostaglandins (PG) E1 and E2 (Tamai *et al*., 2000; Huber *et al*., 2007). GTEx data show that *SLCO3A1* is strongly expressed in the spinal cord, substantia nigra, hippocampus, hypothalamus, anterior cingulate cortex, and frontal cortex. *SLCO3A1* is widely expressed in many cells in the brain including pericytes, cells that are integral to the blood-brain barrier, and therefore this protein is likely to have a role in transport of organic anions across the blood-brain barrier (Sweeney *et al.*, 2019). Two splice isoforms of human *SLCO3A1* show differences in localization with the major isoform being expressed in the basolateral plasma membrane of the choroid plexus and in the grey matter of the frontal cortex, whereas the minor isoform is expressed in the apical pole of epithelial cells of the choroid plexus and white matter of the frontal cortex (Huber *et al*., 2007).

The T allele of rs9806493, a risk variant in our GWAS, increases the expression of *SLCO3A1* which is predicted to lead to increased uptake of hormones including PGE1, PGE2, T4, and vasopressin. Increased levels of PGE1, PGE2, and T4 have been reported in ASPD. For example, thyroid hormones have been suggested to influence the development of aggression in antisocial ASPD patients (Evrensel *et al.*, 2016). In that study, as T3 and T4 levels increased, the aggression scores in ASPD patients also increased (Evrensel *et al.*, 2016). An early study that investigated PG in alcoholic and ASPD patients found that concentrations of PGE1 and PGE2 are higher in ASPD patients than in male controls (Virkkunen *et al.*, 1987). Vasopressin influences social responses including empathy and ASPD is associated with deficiencies in affective empathy (Tabak *et al*., 2015) However, the exact impact of vasopressin on ASPD or antisocial behaviour or both remains unclear. Taken together, we present evidence that genetic variation in the *SLCO3A1* gene may confer risk for ASPD via altered hormone levels.

ASPD shows considerable comorbidity with other psychiatric disorders (Abram *et al.*, 2015; Tielbeek *et al.*, 2018b). The polygenic risk score analysis undertaken in the present study provides further evidence that common genetic loci underlie the risk for ASPD and other complex traits including smoking, alcohol use frequency, PTSD, ADHD, reproductive traits, and educational attainment. These findings were consistent with previous genetic correlation study of antisocial behaviours, which showed that antisocial behaviour was significantly correlated with lifetime cannabis use and cigarettes smoked per day (Tielbeek *et al.*, 2018b). Tielbeek *et al*. examined the genetic correlations of antisocial behaviour and life-history traits and found that genetic risk of antisocial behaviour are positively correlated with higher reproductive traits and negatively correlated with delayed reproductive traits (Tielbeek *et al*., 2018a). This is consistent the PRS analyses in the present study, which showed that showed ASPD risk is negatively correlated with age at the first live birth (in females) and age at first live birth (in males; Fig. 3 & 4). Interestingly we observed a negative genetic correlation with ASPD and alcohol frequency. This finding is somewhat counter intuitive but may reflect the differences in genetic architecture between alcohol dependence/harmful alcohol use and alcohol consumption *per se* (Kranzler *et al*., 2019). Further work in samples of ASPD subjects that do not have a diagnosis of alcohol dependence/harmful alcohol use should allow more precise analysis of these findings. The evidence from our PRS analysis provides further support that ASPD is a highly polygenic disorder that shares genetic risk loci with other psychiatric and neurodevelopmental disorders.

### Limitations

The power of this study was restricted by sample size despite the use of much larger samples than in previous studies. Moreover, this study only used subjects of European ancestry which limits the generalizability of the findings. The results from this study need replication in larger cohorts.

### Conclusion

The study has shown that use of a consistent measure of ASPD diagnostic criteria is a useful approach when exploring associated risk loci. It identified a genome-wide significant association between ASPD criterion score and the *SLCO3A1* gene, which may play a role in the risk for ASPD by regulating hormones levels. This study also provided evidence that ASPD is a polygenic disorder that shares genetic risks with other complex traits.

## Acknowledgements

Supported also by NIH (NIAAA) no. P50 AA12870 (to J.G.), a NARSAD Young Investigator Grant from the Brain & Behavior Research Foundation (to H.Z). UCL cases were collected with UK Medical Research Council project grants nos. G9623693N, G0500791, G0701007 and G1000708, and with support from the National Institutes for Health Research (NIHR) Mental Health Research Network (MHRN). Genotyping of the UCL case samples was funded by the NIHR BRC. M.M.N. and F.D. were supported through grants SysMedAlcoholism (01ZX1611B) and SysMedSUDs by the German Federal Ministry of Education and Research (BMBF) within the e: Med programme. W.L. is supported by China Scholarship Council (CSC) for his PhD studies. N.B. and A.M. are supported by the University College London Hospitals NHS Foundation Trust NIHR BRC.

### Conflicts of interest

H.R.K. is a member of advisory boards for Dicerna Pharmaceuticals, Sophrosyne Pharmaceuticals, Clearmind Medicine, and Enthion Pharmaceuticals; a consultant to Sobrera Pharmaceuticals; the recipient of research funding and medication supplies for an investigator-initiated study from Alkermes. a member of the American Society of Clinical Psychopharmacology’s Alcohol Clinical Trials Initiative, which was supported in the last three years by Alkermes, Dicerna, Ethypharm, Lundbeck, Mitsubishi, and Otsuka. H.R.K. and J.G. are holders of U.S. patent 10,900,082 titled: “Genotype-guided dosing of opioid agonists,” issued 26 January 2021. For the remaining authors, there are no conflicts of interest.

## Supplementary Material




