# Supplementary material for: Genome-wide association study of antisocial personality disorder diagnostic criteria provides evidence for shared risk factors across disorders
Source: Psychiatr Genet. 2023 Sep 19;33(6):233–42. doi: 10.1097/YPG.0000000000000352 (PMC10635348; doi:10.1097/YPG.0000000000000352)
Supplement: Supplementary file 1 [file pg-33-233-s001.pdf]

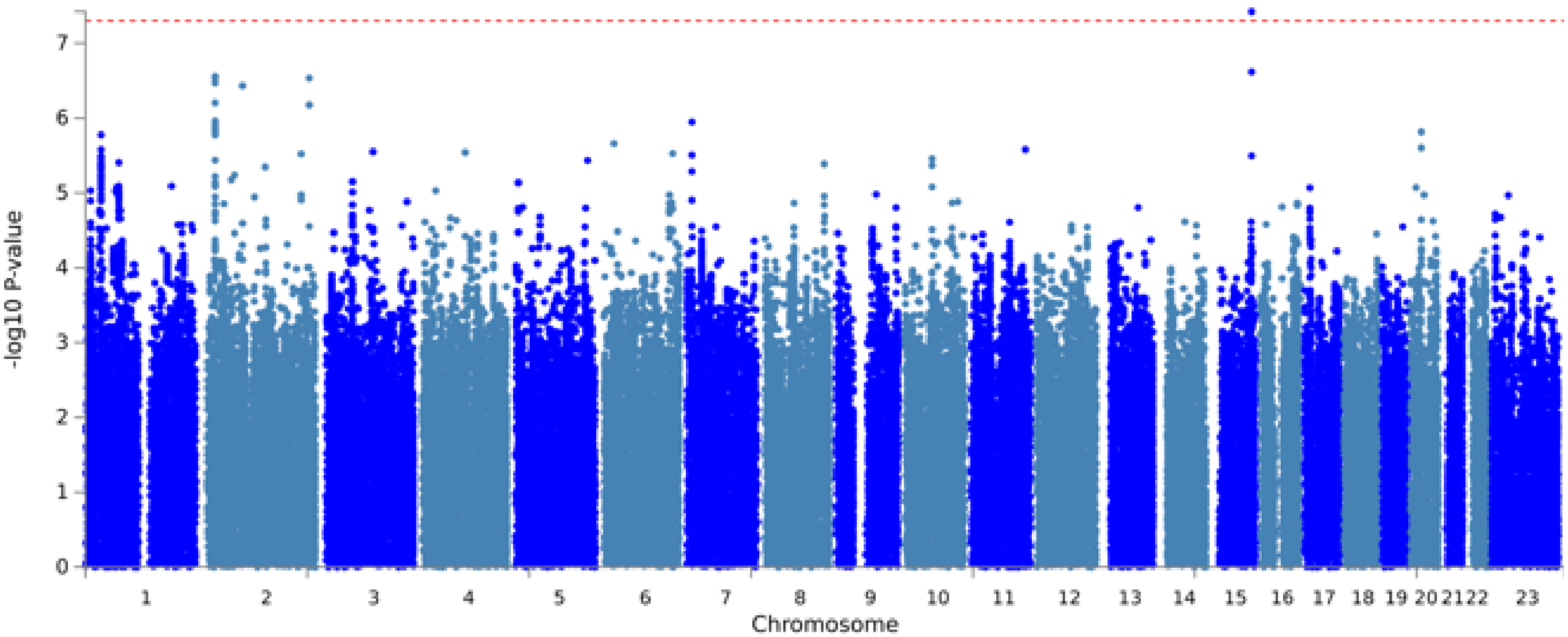

Supplementary Figure 1. GWAS Meta-analysis of quantitative ASPD symptoms in the UCL and Yale-Penn samples.

The positions of the autosomal and chromosome X SNPs are shown on the X axis and the -log10 of the significance values from the meta-analysis of quantitative ASPD symptoms in the UCL and Yale-Penn samples is shown on the Y axis. Genome-wide significance (P = 5x 10^{-8}) is shown by the dotted red line

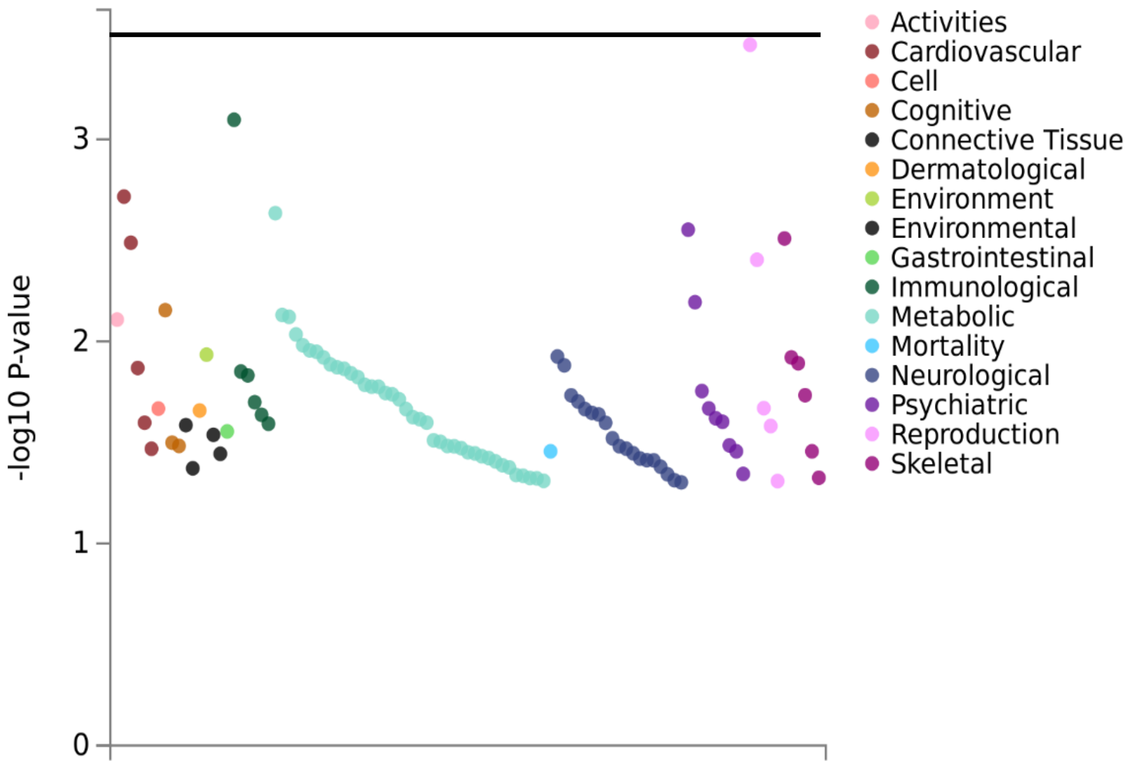

Supplementary Figure 2: rs9806493 PheWAS plot

Phenome-wide association analysis for rs9806493 from 103 GWASs. The results are sorted by domain and P-value. The Bonferroni corrected P-value threshold is 4.85×10^{-4} (horizontal black line). The data for this plot is shown in supplementary table 11.

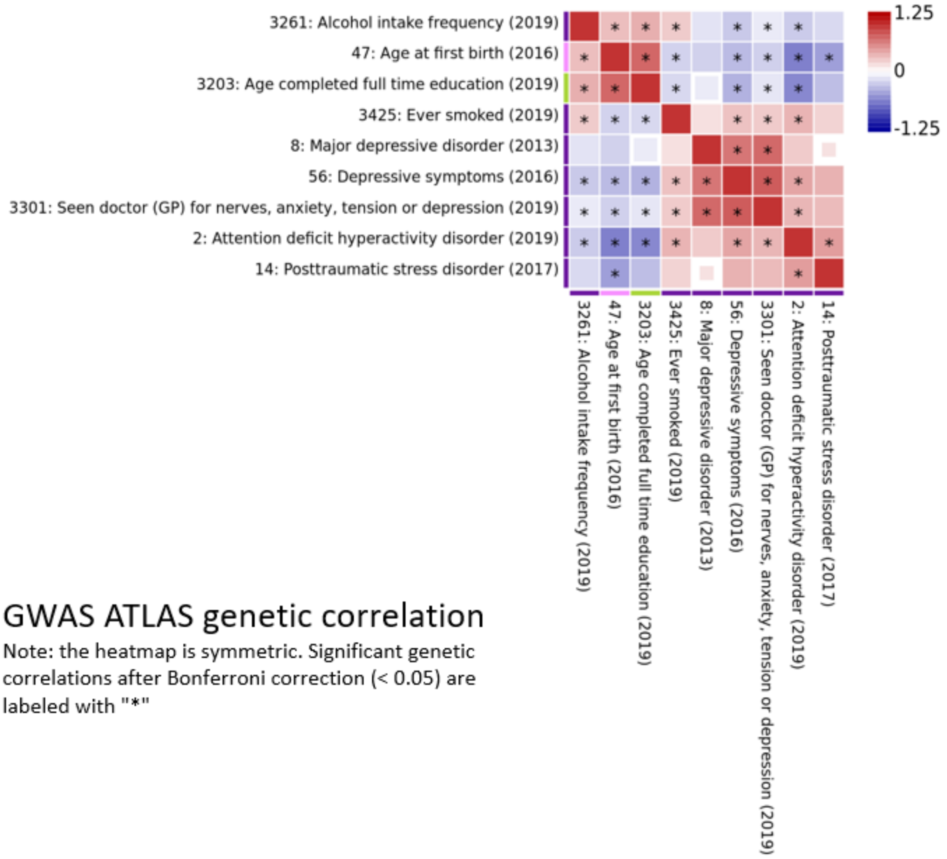

Supplementary Figure 3: genetic correlation heatmap for related traits.
Results were generated from GWAS ATLAS platform (https://atlas.ctglab.nl/traitDB)

Supplementary Material 1

DSM-IV CRITERIA for Conduct Disorder and Antisocial Personality Disorder 1, 26

Evidence of Conduct Disorder with onset before age 15 as indicated by a history of three or more of the following:

1. Often bullied, threatened, or intimidated others
2. Often initiated physical fights
3. Used a weapon that can cause serious physical harm to others (e.g., a bat, brick, broken bottle, knife, gun)
4. Were physically cruel to people
5. Were physically cruel to animals
6. Stole with confrontation of a victim (e.g., mugging, purse-snatching, extortion, armed robbery)
7. Forced someone into sexual activity
8. Deliberately engaged in fire setting with the intention of causing serious damage
9. Deliberately destroyed other's property (other than by fire-setting)
10. Broke into someone else's house, building, or car
11. Often lied to obtain goods or favours or to avoid obligations (i.e., "cons" others)
12. Stole items of nontrivial value without confrontation of a victim (e.g., shoplifting, but without breaking and entering; forgery)
13. Often stayed out at night despite parental prohibitions, beginning before age 13 years
14. Ran away from home overnight at least twice while living in parental or parental surrogate home (or once without returning for a lengthy period
15. Were often truant from school, beginning before age 13

Evidence of Antisocial Personality Disorder indicated by Evidence of Conduct disorder (see above) and a pervasive pattern of disregard for and violation of the rights of others occurring since age 15 years, as indicated by three or more of the following. Note that subjects must be aged 18 or over to receive a diagnosis of Antisocial Personality Disorder:

1. Failure to conform to social norms / lawful behaviours by arrestable deed. Evidenced by:
    a. Steal money or things from family or friends
    b. Break into someone's home, car, or building
    c. Steal money or property by using force or threatening
    d. Set fires on purpose (in order to cause damage)
    e. Damage property on purpose

   f. Deliberately write bad cheques, receive/sell/buy stolen goods, sell drugs, run numbers (illegal gambling), get paid for having sex with someone, or find customers for male or female prostitutes?

2. Deceitfulness. Evidenced by:
   a. Tell a lot of lies, lie to get out of trouble or use an alias
   b. Often cheat (on schoolwork, exams, work, taxes)
   c. Enjoy conning people to get one over on them 3+ times?

3. Impulsivity or failure to plan ahead. Evidenced by:
   a. Not provided financial support for your family when you were supposed to
   b. Leave young children under 6 alone while you were out doing something else
   c. Run out of money for food for the family because you had spent it on yourself or going out (2+ times)?

4. Irritable and aggressive. Evidenced by:
   a. Start fights 3+ times
   b. Often hit or assault others
   c. Injure someone on purpose
   d. Use a weapon (not as part of work or to defend self/others)
   e. Force someone into sexual activity?

5. Reckless disregard for safety of self or others. Evidenced by:
   a. Left young children under 6 at home alone
   b. Had unprotected sex (without a condom) with someone you believed could give you a disease, or when you had a disease that could be spread that way
   c. Often taken chances where you or someone else might get physically hurt (fireworks, guns, car racing, etc)?

6. Consistent irresponsibility in work behaviour or financial obligations. Evidenced by:
   a. Failed to pay debts or take care of financial responsibilities

b. Have often not provided financial support for your family when you were supposed to
c. Were frequently late for work, or without a job for 6 months or more in the last 5 years?

7. Lack of remorse, being indifferent to or rationalizing having hurt, mistreated, or stolen from another. Evidenced by:
   a. Often ignored the feelings of others in order to do what you wanted
   b. Often felt that others were to blame for your mistakes
   c. Were never faithful to a partner for more than 1 year?
